# Transcriptome Analysis of Protein Kinase MoCK2, which Affects Acetyl-CoA Metabolism and Import of CK2-Interacting Mitochondrial Proteins into Mitochondria in the Rice Blast Fungus Magnaporthe oryzae

**DOI:** 10.1128/spectrum.03042-22

**Published:** 2022-10-18

**Authors:** Lianhu Zhang, Chonglei Shan, Yifan Zhang, Wenjing Miao, Xiaoli Bing, Weigang Kuang, Zonghua Wang, Ruqiang Cui, Stefan Olsson

**Affiliations:** a College of Agronomy, Jiangxi Agricultural University, Nanchang, Jiangxi, China; b Key Laboratory of Crop Physiology, Ecology and Genetic Breeding, Ministry of Education, Jiangxi Agricultural University, Nanchang, Jiangxi, China; c College of Bioscience and Bioengineering, Jiangxi Agricultural University, Nanchang, Jiangxi, China; d State Key Laboratory for Ecological Pest Control of Fujian and Taiwan Crops, College of Plant Protection, Fujian Agriculture and Forestry University, Fuzhou, Fujian, China; e Department of Entomology, Nanjing Agricultural University, Nanjing, Jiangsu, China; University of Molise

**Keywords:** *Magnaporthe oryzae*, MoCK2, transcriptome analysis, acetyl-CoA, mitochondria

## Abstract

The rice pathogen Magnaporthe oryzae causes severe losses to rice production. Previous studies have shown that the protein kinase MoCK2 is essential for pathogenesis, and this ubiquitous eukaryotic protein kinase might affect several processes in the fungus that are needed for infection. To better understand which cellular processes are affected by MoCK2 activity, we performed a detailed transcriptome sequencing analysis of deletions of the MoCK2 b1 and b2 components in relation to the background strain Ku80 and connected this analysis with the abundance of substrates for proteins in a previous pulldown of the essential CKa subunit of CK2 to estimate the effects on proteins directly interacting with CK2. The results showed that MoCK2 seriously affected carbohydrate metabolism, fatty acid metabolism, amino acid metabolism, and the related transporters and reduced acetyl-CoA production. CK2 phosphorylation can affect the folding of proteins and especially the effective formation of protein complexes by intrinsically disordered or mitochondrial import by destabilizing soluble alpha helices. The upregulated genes found in the pulldown of the b1 and b2 mutants indicate that proteins directly interacting with CK2 are compensatorily upregulated depending on their pulldown. A similar correlation was found for mitochondrial proteins. Taken together, the classes of proteins and the changes in regulation in the b1 and b2 mutants suggest that CK2 has a central role in mitochondrial metabolism, secondary metabolism, and reactive oxygen species (ROS) resistance, in addition to its previously suggested role in the formation of new ribosomes, all of which are processes central to efficient nonself responses as innate immunity.

**IMPORTANCE** The protein kinase CK2 is highly expressed and essential for plants, animals, and fungi, affecting fatty acid-related metabolism. In addition, it directly affects the import of essential mitochondrial proteins into mitochondria. These effects mean that CK2 is essential for lipid metabolism and mitochondrial function and, as shown previously, is crucial for making new translation machinery proteins. Taken together, our new results combined with previously reported results indicate that CK2 is an essential protein necessary for the capacities to launch efficient innate immunity responses and withstand the negative effects of such responses necessary for general resistance against invading bacteria and viruses as well as to interact with plants, withstand plant immunity responses, and kill plant cells.

## INTRODUCTION

The protein kinase CK2, highly expressed and essential for plants, animals, and fungi, affects fatty acid-related metabolism and mitochondrial proteins, making it essential for the capacity to launch efficient innate immunity responses and plant pathogenicity.

Rice is the most important food crop in the world. Most people in the world have rice as one of their staple foods. Thus, rice production plays a vital role in world food security (1). However, huge losses are caused by diseases and pests every year. Among these diseases, rice blast is the most important, seriously affecting rice yields and quality (2), making the prevention, damage limitation, and control of rice blast infections essential to secure the food supply. Presently, the use of resistant plant varieties is the most common method to prevent and control the occurrence of this disease (3). However, with the common practice of crop monoculture of plant cultivars and the dynamic genetic and epigenetic changes in the physiological races of pathogens, the loss of resistance against pathogens of plant cultivars is a constant problem and threat (4). Therefore, increased basic knowledge of the mechanism of pathogenicity of the rice blast fungus is another important road toward the goal of preventing, limiting damage from, and hindering disease epidemics with devastating consequences.

Rice blast disease is caused by the fungus Magnaporthe oryzae (5). The infection develops as follows. Conidia are spread by wind, attach to rice leaves, and germinate to form a germ tube. The tip of the germ tube expands to form an appressorium (6). The deposition of melanin on the cell wall makes it strong, and, together with the accumulation of a large amount of glycerol in the appressorium, a huge turgor develops (7). The maturation of the appressorium is accompanied by the autophagy of conidial cells and nuclear mitotic divisions in the germ tube (8, 9). A penetration peg is formed at the bottom of the mature appressorium, which pierces rice epidermal cells to complete the infection and enter the plant epidermal cell interior (10). Under suitable local conditions for pathogen invasion, rice leaves form disease spots after 5 to 7 days and then produce new conidia. Conidia spread and start a new disease cycle if they are spread to uninfected plants by the power of wind and rain (11). Previous research has shown that the infection process of M. oryzae is regulated by a series of signals, such as through the cAMP pathway and the mitogen-active protein kinase (MAPK) pathway, affecting the production and germination of conidia and the formation of and infection by appressoria (12, 13). Deletion mutants of some important genes, such as the genes encoding protein kinase A (PKA) (12, 13) and the septin proteins involved in septal pore formation and dynamics (14), seriously hindered the infection and pathogenicity of M. oryzae. The protein kinase MoCK2 holoenzyme is an enzyme complex containing two catalytic subunits and two regulatory subunits. The catalytic subunit, named MoCKa, is encoded by one gene, MGG_03696. Two different genes, MGG_00446 and MGG_05651, encode the two regulatory subunits named MoCKb1 and MoCKb2, respectively. MoCKa deletion is lethal, and mutants cannot be obtained. The deletion mutants of the two regulatory subunits MoCKb1 and MoCKb2 showed severe phenotypic defects *in vitro* (here, we use b1 for the deletion mutant of MoCKb1 and b2 for the deletion mutant of MoCKb2). Both the b1 and b2 mutants grew slowly, showed severely decreased spore production, and lost pathogenicity. This was accompanied by a complete loss of the capacity for infection of rice leaves, indicating that the MoCK2 holoenzyme supplies the pathogen with important functions needed for its pathogenic life cycle (15). We further found thousands of proteins interacting with MoCKa in a pulldown experiment. That analysis showed that the phosphorylation of MoCK2 and the accompanying dephosphorylation have important functions in temporarily destabilizing alpha helices, allowing the normal formation of IDP (intrinsically disordered protein) protein-protein and protein-polymer interactions and the formation of new ribosomes (16).

Based on this, we aimed to more accurately understand how the deletions of the MoCK2 components affect the activities of M. oryzae by transcriptome analysis of the b1 and b2 mutants compared with the background strain Ku80 under *in vitro* conditions. Transcriptome sequencing employing second-generation sequencing was performed to analyze the transcriptome differences between the b1 and b2 mutants and the background strain Ku80 to get an idea of which processes are affected in the mutants and why we observed particular phenotypes.

Transcriptome sequencing technology has been widely used for scientific research, including for the interaction between rice and M. oryzae. Gene expression changes in susceptible and resistant rice varieties infected by M. oryzae have been analyzed by transcriptome sequencing and play important guiding roles in the development of new rice varieties (17).

Currently, RNA-seq is an often-used convenient means to obtain indications of extended roles of genes and a comprehensive understanding of genes in the whole genome affected by a target gene’s direct and indirect activities. It is then generally assumed that most of the affected genes will lack expression in the mutant compared with the wild type with the intact genes. There can, however, be compensatory upregulation in other genes that can hide the full effects of such deletions (18). In addition, we want to investigate the transcriptional effects of the CKb mutations on genes for the specific proteins found in the previous pulldown that directly interact with CKa, as our previous work indicated that these proteins should help form proper interactions with other proteins by CK2 activity. Compensatory upregulation of the corresponding genes can be expected to compensate for inefficient CK2 function in the b1 and b2 mutants if CK2 facilitates IDP-forming functional interactions; thus, this is specially investigated.

In this study, transcriptome sequencing showed that the deletion of the protein kinase MoCK2 of M. oryzae seriously affected cellular metabolism to reduce acetyl-CoA production. Compared with the background strain Ku80, the expression of genes involved in carbohydrate metabolism, fatty acid metabolism, and amino acid metabolism in the b1 and b2 mutants was downregulated, thus likely affecting acetyl-CoA production, which seriously negatively affects the processes of energy and substrate metabolism, causing the observed phenotypic defects in the deletion mutants. Several genes encoding proteins found to interact with CKa in the previous pulldown study were upregulated. This compensatory upregulation indicates that both CKb1 and -2 are important for many aspects of fungal growth and pathogenesis. However, CKb1 is likely more important for pathogenesis than CKb2 as it seems more important for fast translational responses.

## RESULTS

### Changes in gene expression due to CKb deletion.

**FIG 1:**
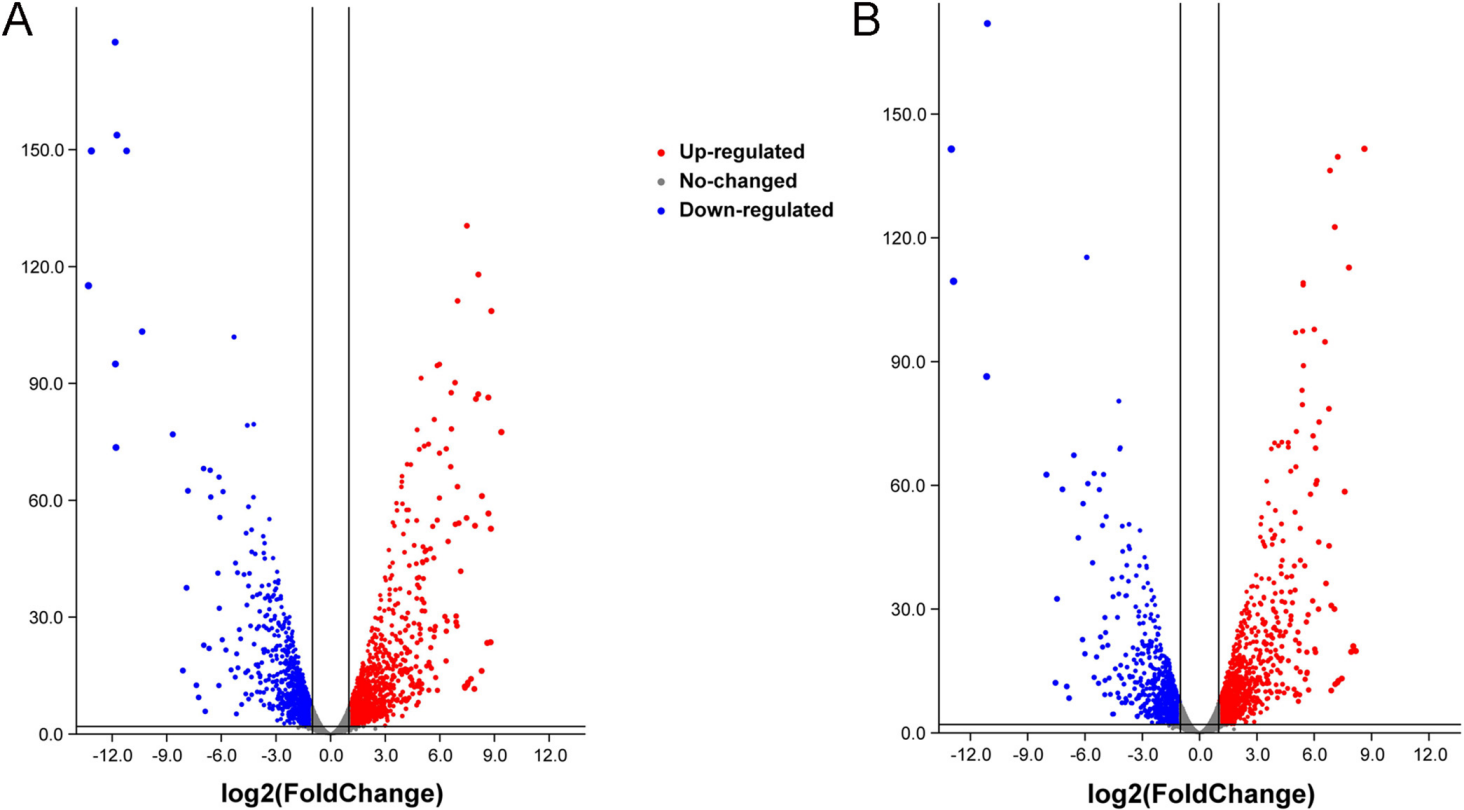
Differentially expressed genes (DEGs) in the b1 mutant (A) and the b2 mutant (B) compared with background strain Ku80. The blue points show the downregulated expression of genes in both mutants compared with background strain Ku80. The red points show the upregulated expression of genes in both mutants compared with background strain Ku80. We considered genes with *P* values of <0.05 and absolute fold change values of >2 to be significantly differentially expressed genes.

In this study, we considered the genes with *P* values of <0.05 and absolute fold change values of >2 to be significantly differentially expressed genes (DEGs). These are relatively strong criteria because we are interested primarily in “downstream effector genes” with a direct effect on phenotypes since the changes in the expression of these genes could explain why we observe the deletion phenotypes in culture. These criteria identified 1,297 differentially upregulated genes and 1,025 downregulated genes for the b1 mutant compared with the background strain Ku80. For the b2 mutant, the values were slightly lower, with 1,189 differentially upregulated genes and 862 differentially downregulated genes (Fig. 1; see also Table S1, sheets 7 to 12, in the supplemental material).

**FIG 2:**
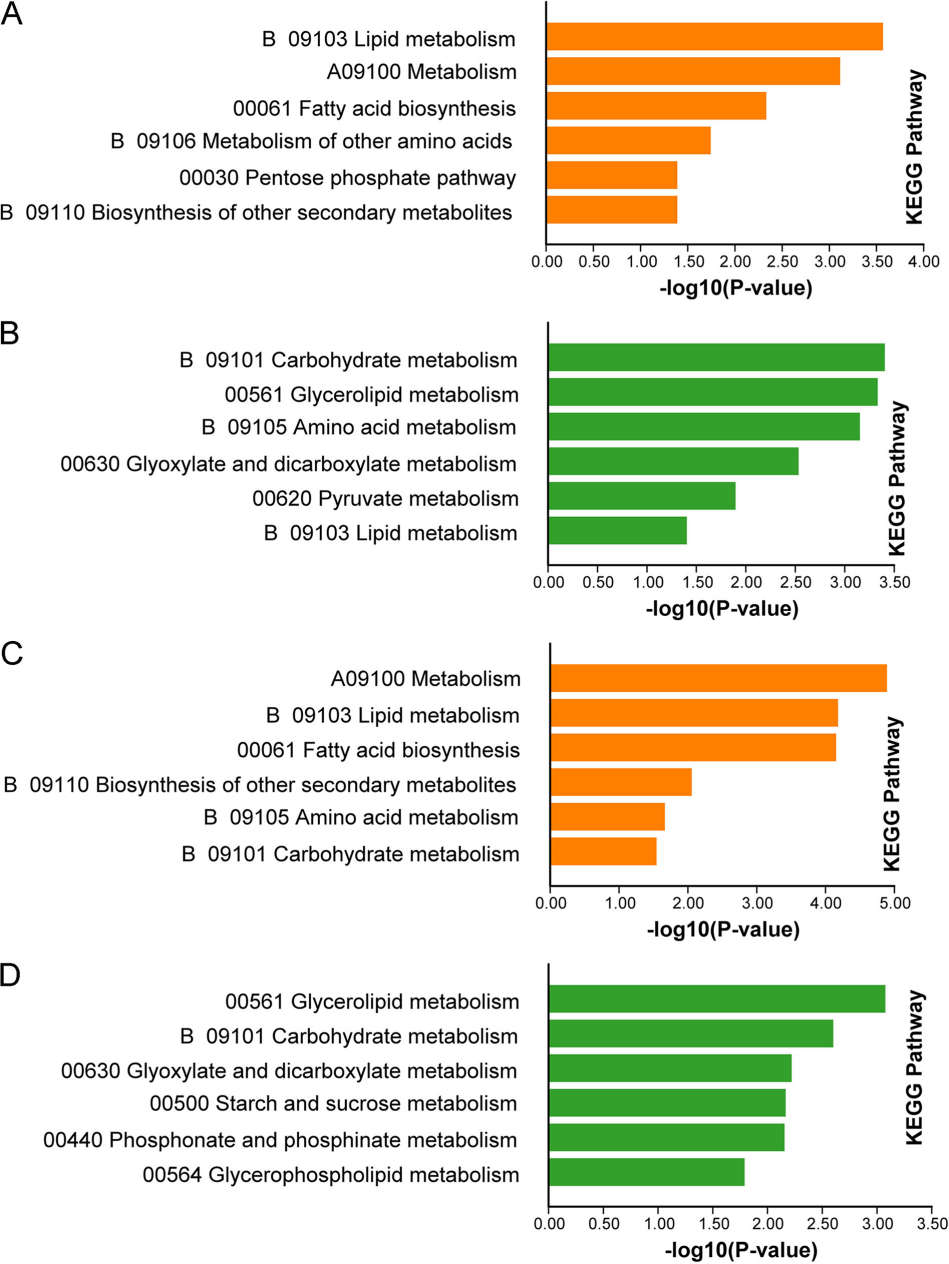
Some results of KEGG enrichment analysis of DEGs in the b1 and b2 mutants compared with background strain Ku80. (A) KEGG enrichment of upregulated genes in the b1 mutant compared with Ku80; (B) KEGG enrichment of downregulated genes in the b1 mutant compared with Ku80; (C) KEGG enrichment of upregulated genes in the b2 mutant compared with Ku80; (D) KEGG enrichment of downregulated genes in the b2 mutant compared with Ku80. The *P* values of the KEGG enrichment terms shown here are <0.05.

**FIG 3:**
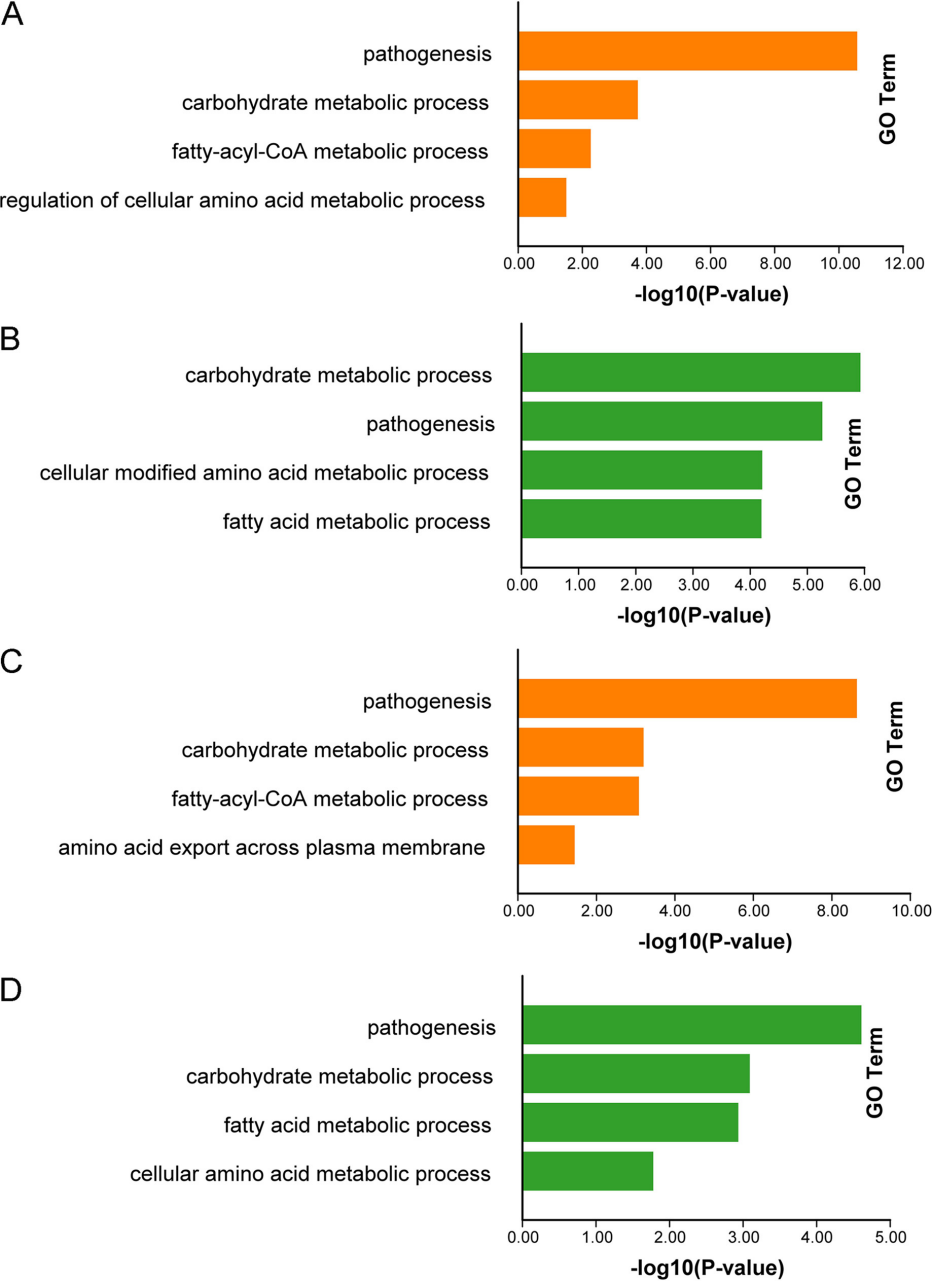
Some results of GO enrichment analysis of DEGs in the b1 and b2 mutants compared with background strain Ku80. (A) GO enrichment of upregulated genes in the b1 mutant compared with Ku80; (B) GO enrichment of downregulated genes in the b1 mutant compared with Ku80; (C) GO enrichment of upregulated genes in the b2 mutant compared with Ku80; (D) GO enrichment of downregulated genes in the b2 mutant compared with Ku80. The *P* values of the GO enrichment terms shown here are <0.05.

In order to understand the functions of DEGs between the b1 and b2 mutants and background strain Ku80, KEGG (Kyoto Encyclopedia of Genes and Genomes) (19) and GO (Gene Ontology) analyses were used to analyze the DEGs using TBtools (20). We focused mainly on the genes downregulated in the mutants since these are the ones that would be the most positively affected by intact CK2 function. KEGG analysis of DEGs between the b1 and b2 mutants and background strain Ku80 showed that the deletion of protein kinase MoCK2 components b1 and b2 affected cell metabolism. Differentially up-regulated or down-regulated genes were significantly enriched in metabolism, especially cell carbohydrate metabolism, lipid metabolism, protein (amino acid) metabolism, and secondary metabolism (Fig. 2 and Table S2, sheets 1 to 4). The numbers of genes with GO annotations for the upregulated and downregulated DEGs in the b1 mutant were 482 and 552, respectively. Similarly, the b2 mutant showed 506 and 395 GO-annotated DEGs, respectively. The significantly enriched GO categories also imply involvement in carbohydrate metabolism, lipid metabolism, and protein (amino acid) metabolism, confirming what was found for the KEGG-classified proteins (Fig. 3 and Table S2, sheets 5 to 12). From the results of the KEGG and GO analyses, we noted that protein kinase MoCK2 played an essential role in the growth and development of M. oryzae and that the deletion of MoCK2 component affected critical metabolic processes that can result in the obvious phenotypic defects observed previously (15).

### Acetyl-CoA synthesis is affected in the deletion mutants.

Acetyl-CoA is a link between various types of metabolism (21). Therefore, we focused on which of these could be affected by the protein kinase MoCK2.

The primary sources of acetyl-CoA in a cell are carbohydrate catabolism, including glycolysis and the dehydrogenation of pyruvate; fatty acid β-oxidation; and amino acid oxidative respiration. The central role of acetyl-CoA in catabolism is to convert chemical energy through the cyclic oxidation of tricarboxylic acid, or fatty acids, to more generally useful energy intermediates (ATP, NADH, NADPH, and electrochemical membrane potential) and/or participate in anabolic pathways by supplying biosynthesis “building blocks” (21) (Fig. S3). Thus, we looked at the changes in the expression of genes involved in acetyl-CoA metabolism to determine whether MoCK2 could affect acetyl-CoA metabolism.

**FIG 4:**
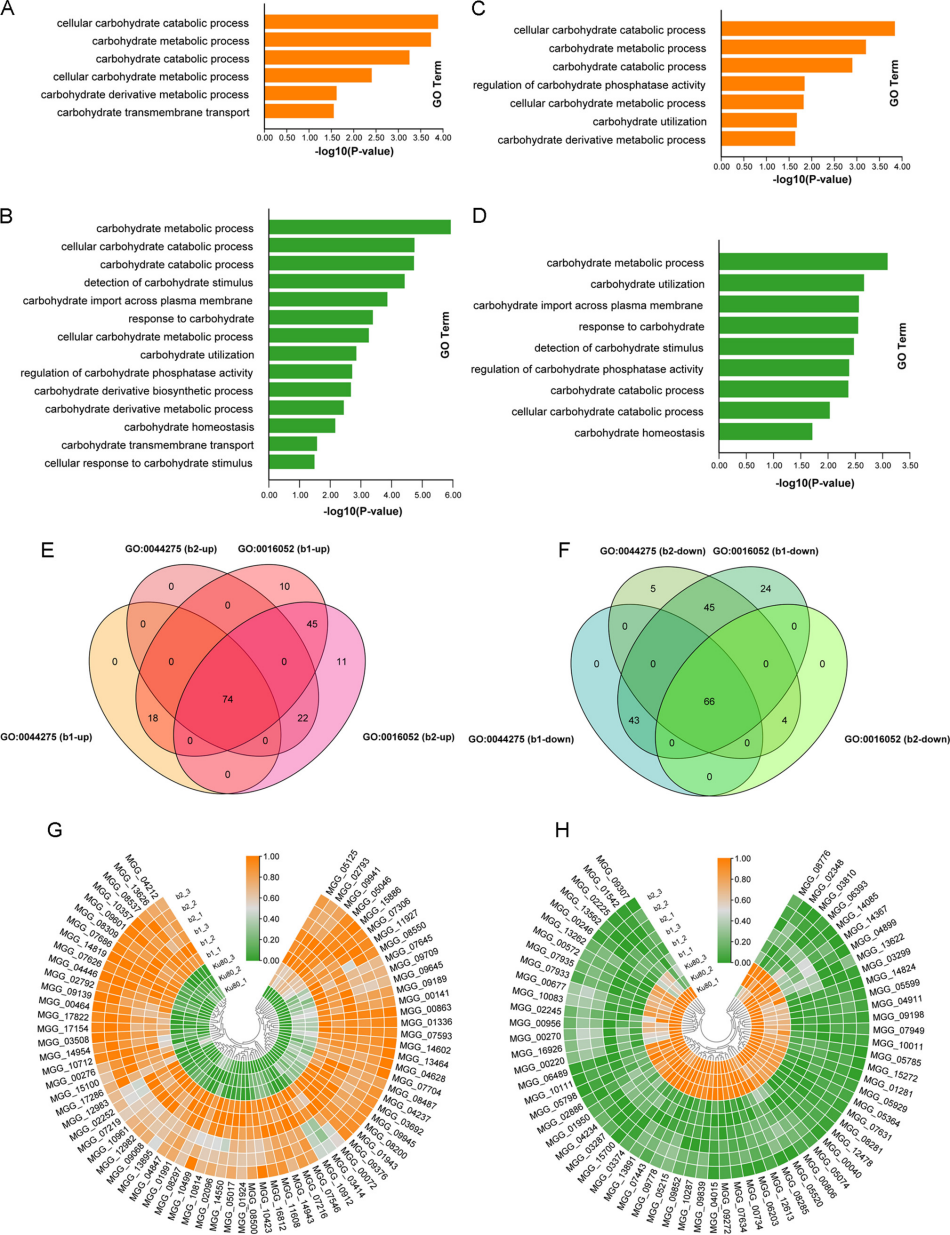
GO enrichment and expression heat map of some genes involved in the carbohydrate metabolic process in the b1 and b2 mutants. (A) GO enrichment of upregulated genes involved in the carbohydrate metabolic process in the b1 mutant; (B) GO enrichment of downregulated genes involved in the carbohydrate metabolic process in the b1 mutant; (C) GO enrichment of upregulated genes involved in the carbohydrate metabolic process in the b2 mutant; (D) GO enrichment of downregulated genes involved in the carbohydrate metabolic process in the b2 mutant; (E) the number of upregulated genes from both the b1 and b2 mutants enriched in GO:0044275 and GO:0016052 was 74; (F) the number of downregulated genes from both the b1 and b2 mutants enriched in GO:0044275 and GO:0016052 was 66; (G) heat map indicating the 74 upregulated genes enriched in GO:0044275 and GO:0016052 in the b1 and b2 mutants; (H) heat map indicating the 66 downregulated genes enriched in GO:0044275 and GO:0016052 in the b1 and b2 mutants. Genes showing similar patterns of expression are clustered.

First, we detected genes annotated as belonging to carbohydrate metabolism in both mutants. The results showed that the deletion of MoCK2 components likely seriously affected carbohydrate metabolism in the cells. In the b1 mutant, 226 upregulated DEGs have and annotation for the carbohydrate metabolism pathway (GO:0005975), and 274 downregulated DEGs were significantly enriched for genes annotated as belonging to GO:0005975 (*P* ≤ 0.05). The genes enriched for GO:0005975 accounted for 48% of the DEGs recorded for the b1 mutant. This was similar for the b2 mutant, in that there were 234 upregulated DEGs and 188 genes with downregulated expression significantly enriched for genes annotated as belonging to GO:0005975 (*P* ≤ 0.05). The genes enriched in GO:0005975 accounted for 46% of the DEGs in the b2 mutant (Fig. 4 and Table S3, sheets 1 to 4). The genes involved in carbohydrate catabolism were mainly enriched in two GO terms: the cellular carbon catabolic process (GO:0044275) and the carbon catabolic process (GO:0016052) (Fig. 4A to D and Table S3, sheets 5 and 7). The number of upregulated DEGs from both mutants significantly enriched in GO:0044275 and GO:0016052 was 74 (*P* ≤ 0.05), and the number of downregulated DEGs significantly enriched was 66 (*P* ≤ 0.05) (Fig. 4E and F and Table S3, sheets 6 and 8). Some enzymes in carbohydrate metabolism, such as endoglucanase and glycoside hydrolase, have many encoding genes in cells. Their expression changes also show different trends, where some have upregulated DEGs and some have downregulated DEGs, indicating the complexity of the involvement of CK2 in carbohydrate metabolism.

In addition, the upregulated gene MGG_10423, encoding cellulase, and the downregulated gene MGG_03287, encoding amylase, indicated that MoCK2 had diverse regulatory mechanisms for the catabolism of polysaccharides in M. oryzae. Moreover, some sugar transporters, such as high-affinity glucose transporters, hexose transporters, and the sugar transporter STL1, had an undeniable trend toward downregulation in both mutants, indicating that the deletion of MoCK2 affected the absorption and transport of sugars, resulting in significant changes in the expression of genes related to glucose metabolism, especially catabolism (Fig. 4G and H and Table S3, sheets 6 and 8).

**FIG 5:**
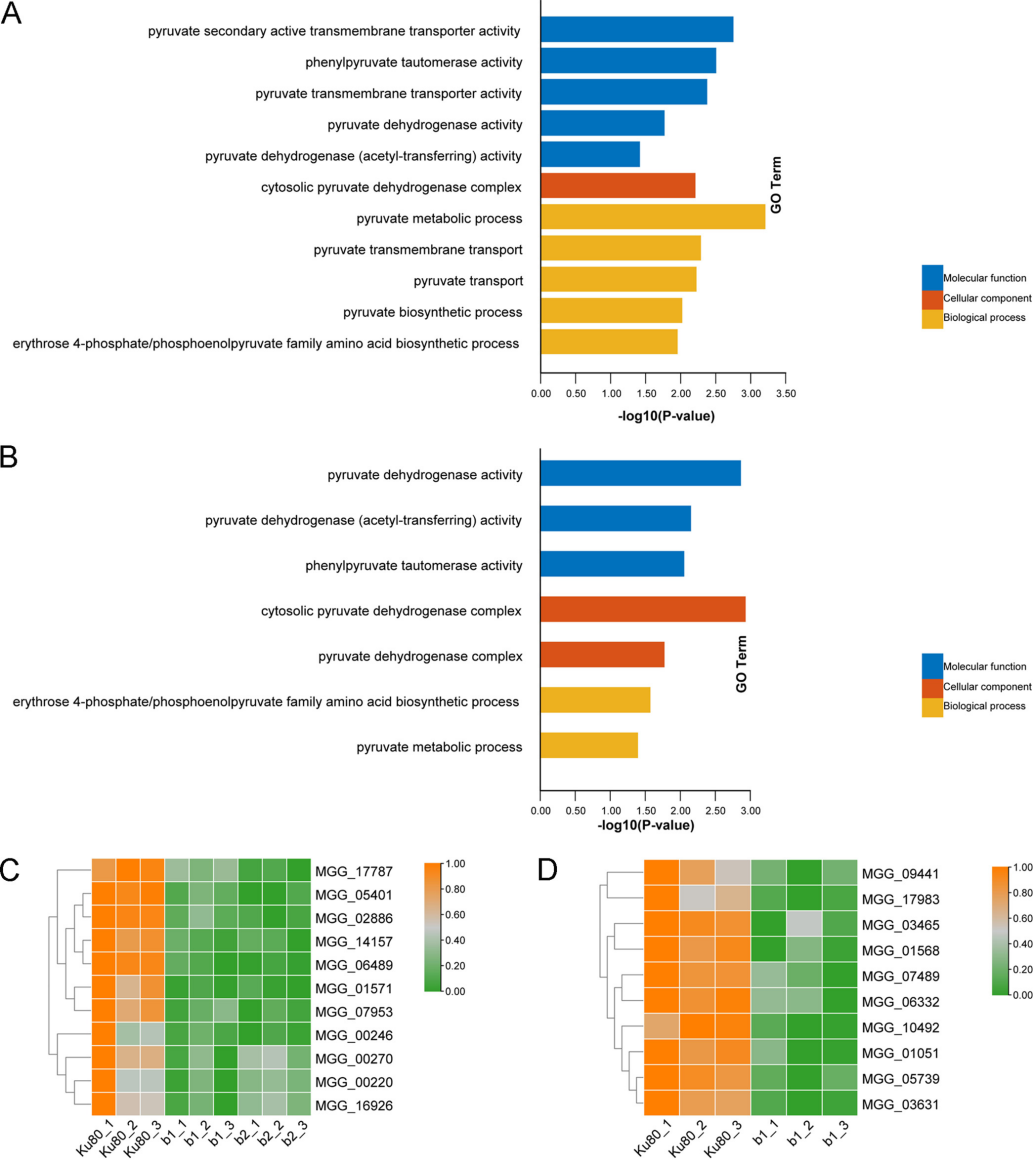
Analysis of gene expression involving pyruvate dehydrogenase in the b1 and b2 mutants. (A) GO enrichment of pyruvate metabolism in the b1 mutant; (B) GO enrichment of pyruvate metabolism in the b2 mutant; (C) heat map showing the expression of genes encoding pyruvate dehydrogenases in the b1 and b2 mutants; (D) heat map showing the expression of the genes encoding pyruvate transmembrane transporters in the b1 mutant. Genes showing similar patterns of expression are clustered.

Glucose is converted into pyruvate through glycolysis and mitochondrial import. Pyruvate in the mitochondria is converted to acetyl-CoA through a series of reactions starting with pyruvate dehydrogenase (22). Therefore, we tested whether the expression of the mitochondrial pyruvate dehydrogenase-encoding gene and the following reactions were affected in the b1 and b2 mutants. The results showed that in the b1 and b2 mutants, 11 downregulated DEGs were significantly enriched for mitochondrial pyruvate dehydrogenase metabolism genes (Fig. 5A to C and Table S4). We also found that the expression of pyruvate transmembrane transporter genes also showed a downward trend in the b1 mutant, indicating that the deletion of MoCK2 could also affect pyruvate transmembrane transport between the cytoplasm and mitochondria or peroxisomes (Fig. 5D and Table S4). The results described above suggested that MoCK2 deletion affected the conversion of pyruvate to acetyl-CoA, affecting downstream metabolism.

The results described above indicate that the deletion of the protein kinase MoCK2 reduced the catabolism of carbohydrates and the activity of pyruvate dehydrogenase in cells and then reduced the content of acetyl-CoA, thus slowing down the metabolism of cells, which was likely an essential cause of the observed phenotypic defects of both mutants (15).

### Acetyl-CoA synthesis from fatty acid metabolism is affected in the deletion mutants.

**FIG 6:**
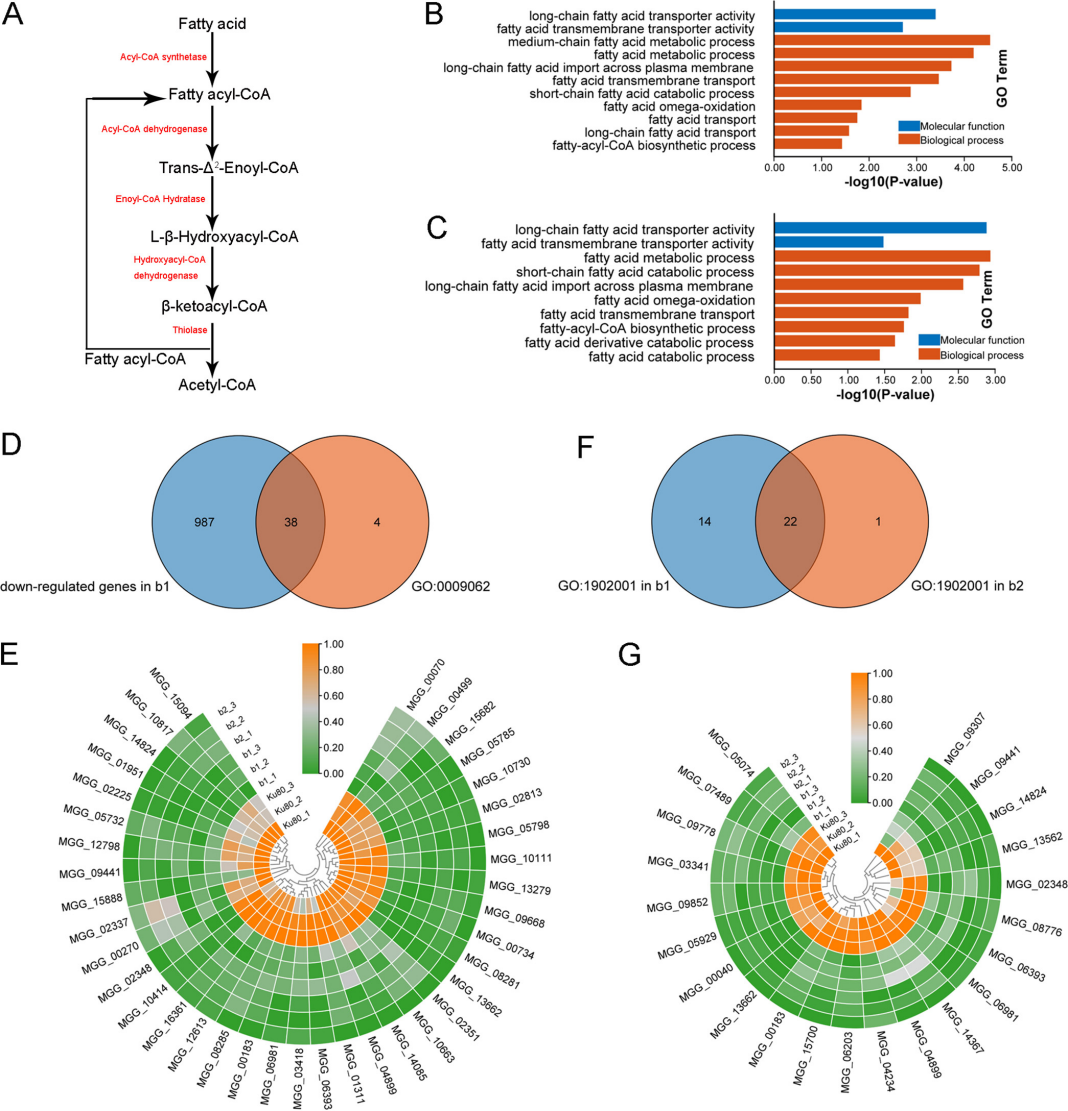
Analysis of gene expression involving fatty acid metabolism in the b1 and b2 mutants. (A) Fatty acid β-oxidation pathway. (B and C) GO enrichment of DEGs in fatty acid metabolism in the b1 (B) and b2 (C) mutants. (D) Venn plot indicating 38 genes enriched in the fatty acid catabolic process (GO:0009062) in the b1 and b2 mutants. (E) Expression of 38 genes enriched in the fatty acid catabolic process (GO:0009062) in the b1 and b2 mutants compared with background strain Ku80. MGG_06918 encodes carnitine acetyltransferase; MGG_03418 encodes acyl-CoA dehydrogenase. (F) Venn plot indicating 22 genes enriched in fatty acid transmembrane transport (GO:1902001) in the b1 and b2 mutants. (G) Expression of 22 genes enriched in fatty acid transmembrane transport (GO:1902001) in the b1 and b2 mutants compared with background strain Ku80. Genes showing similar patterns of expression are clustered.

Fatty acid catabolism, especially β-oxidation, is a significant source of acetyl-CoA, especially in conidia (23) (Fig. 6A), and we detected expression changes in the related genes (Fig. 6B and C and Table S5, sheets 1 to 4). The results showed that 42 downregulated genes in the b2 mutant and 38 downregulated genes in the b1 mutant were enriched in the GO term fatty acid catabolic process (GO:0009062) (Fig. 6D and E and Table S5, sheet 5). Further analysis showed that two critical enzymes, carnitine acetyltransferase, encoded by MGG_06918, and acyl-CoA dehydrogenase, encoded by MGG_03418, were downregulated in both deletion mutants. Carnitine acetyltransferase (24), which transports acetyl-CoA between subcellular compartments, controls the beginning of fatty acid oxidation. Acyl-CoA dehydrogenase converts acyl-CoA to enoyl-CoA and reduces FAD (flavin adenine dinucleotide) to FADH_2_ (reduced flavin adenine dinucleotide) (25, 26). FADH_2_ enters the mitochondrial electron transport chain for oxidation and participates in the generation of the pH gradient over the mitochondrial inner membrane, resulting in ATP being exchanged for ADP and exported to the rest of the cell. The reduced expression of carnitine acetyltransferase and acyl-CoA dehydrogenase likely affects the oxidative degradation of fatty acids, reducing the cellular content of acetyl-CoA and affecting energy conversion metabolism, resulting in cells that do not have enough available energy in suitable forms for driving cellular anabolic processes. Supporting the involvement in fat metabolism, fatty acid transmembrane transporters were enriched (GO:1902001). The number of these genes affected in both mutants was 22 (Fig. 6F and G and Table S5, sheet 6). The decrease in the expression of transporter-encoding genes indicates that the deletion of the protein kinase MoCK2 reduced the transmembrane transport of fatty acids in cells, which can directly affect the rate of fatty acid oxidation.

In conclusion, the deletion of MoCK2 likely affected the production of acetyl-CoA by reducing fatty acid metabolism and intracellular transport. It then affected the subsequent rate of formation of cellular energy “currencies”, as the ATP is needed for growth, maintenance, and the synthesis of extracellular compounds, resulting in the previously observed severe phenotypic defects (15).

### Acetyl-CoA synthesis from amino acid metabolism is affected in the deletion mutants.

**FIG 7:**
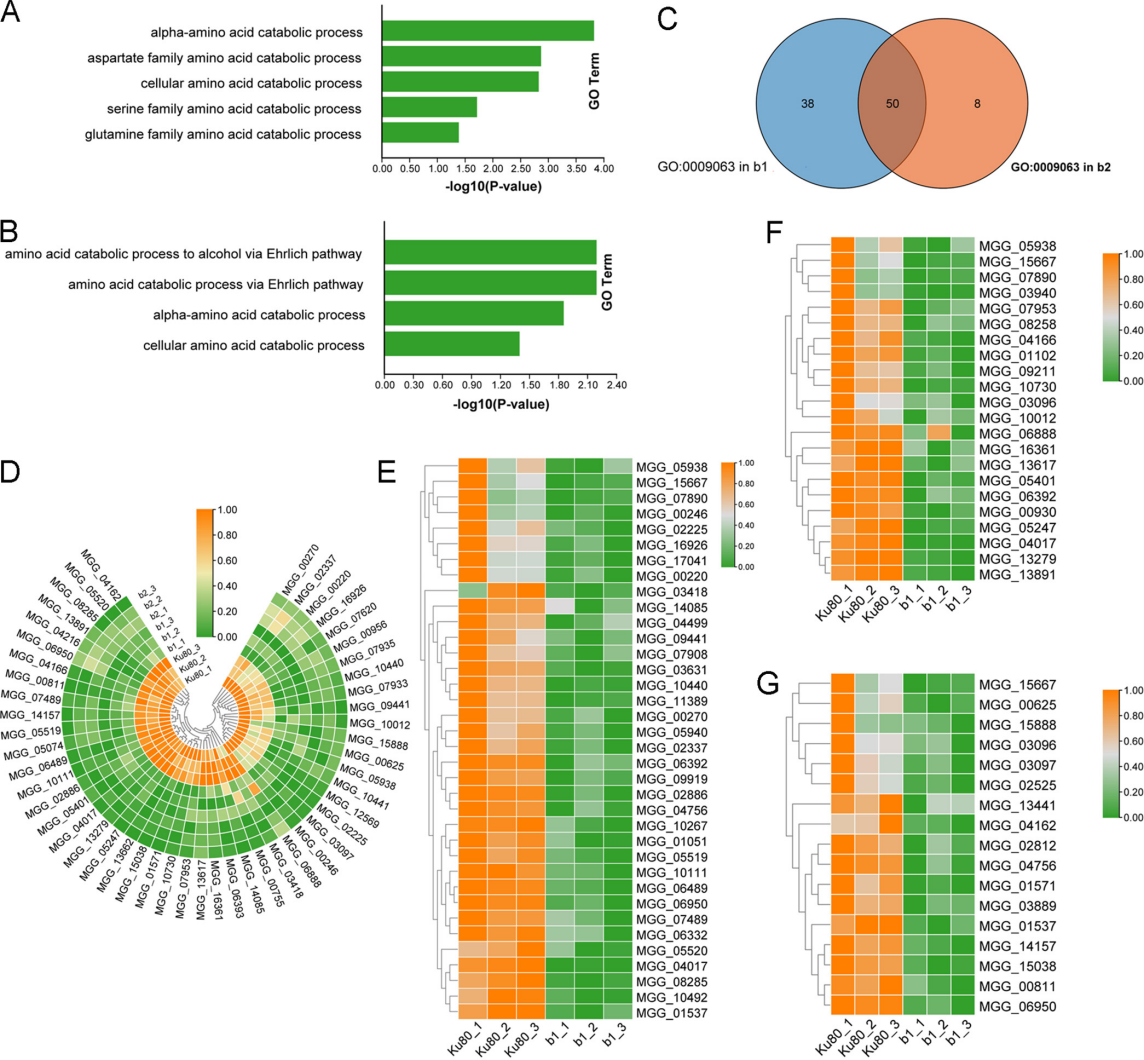
Expression of genes involved in the amino acid catabolic pathway. (A) GO enrichment of the amino acid catabolic process in the b1 mutant; (B) GO enrichment of the amino acid catabolic process in the b2 mutant; (C) genes enriched in GO:0009063 from both the b1 and b2 mutants; (D) heat map showing the expression of 50 genes enriched in GO:0009063 from both the b1 and b2 mutants; (E) heat map showing the expression of genes enriched in the aspartate family amino acid catabolic process (GO:0009068) in the b1 mutant; (F) heat map showing the expression of genes enriched in the glutamine family amino acid catabolic process (GO:0009065) in the b1 mutant; (G) heat map showing the expression of genes enriched in the serine family amino acid catabolic process (GO:0009071) in the b1 mutant. Genes showing similar patterns of expression are clustered.

Amino acid catabolism of alanine, glycine, serine, threonine, and phenylalanine is another significant source of acetyl-CoA. Among the downregulated genes in both mutants, we found GO enrichment terms for amino acid catabolism (Fig. 7A and B and Table S6, sheets 1 to 4). GO:0009063 represents the cellular amino acid catabolic process, in which there were 88 genes downregulated in the b1 mutant and 58 genes downregulated in the b2 mutant. Fifty of these genes were downregulated in both mutants (Fig. 7C and D and Table S6, sheet 5). The deletion of any MoCKb seriously inhibited the catabolism of amino acids. In addition, we found that the aspartate family amino acid catabolic process (GO:0009068), the serine family amino acid catabolic process (GO:0009071), and the glutamine family amino acid catabolic process (GO:0009065) were downregulated in the b1 mutant (Fig. 7E to G and Table S6, sheets 6 to 8). The catabolites of aspartate and glutamine to form α-ketoglutarate and oxaloacetate can directly participate in the tricarboxylic acid cycle. Serine catabolism is another source of acetyl-CoA.

### The expression of transporter-encoding genes is affected in the deletion mutants.

As is stated above in the introduction, the deletion of MoCK2 affected the transmembrane transport of the carbohydrate transporters pyruvate and fatty acids. So we suspected that the blocked transport of various substances was an important reason for the phenotypic defects of the b1 and b2 mutants.

**FIG 8:**
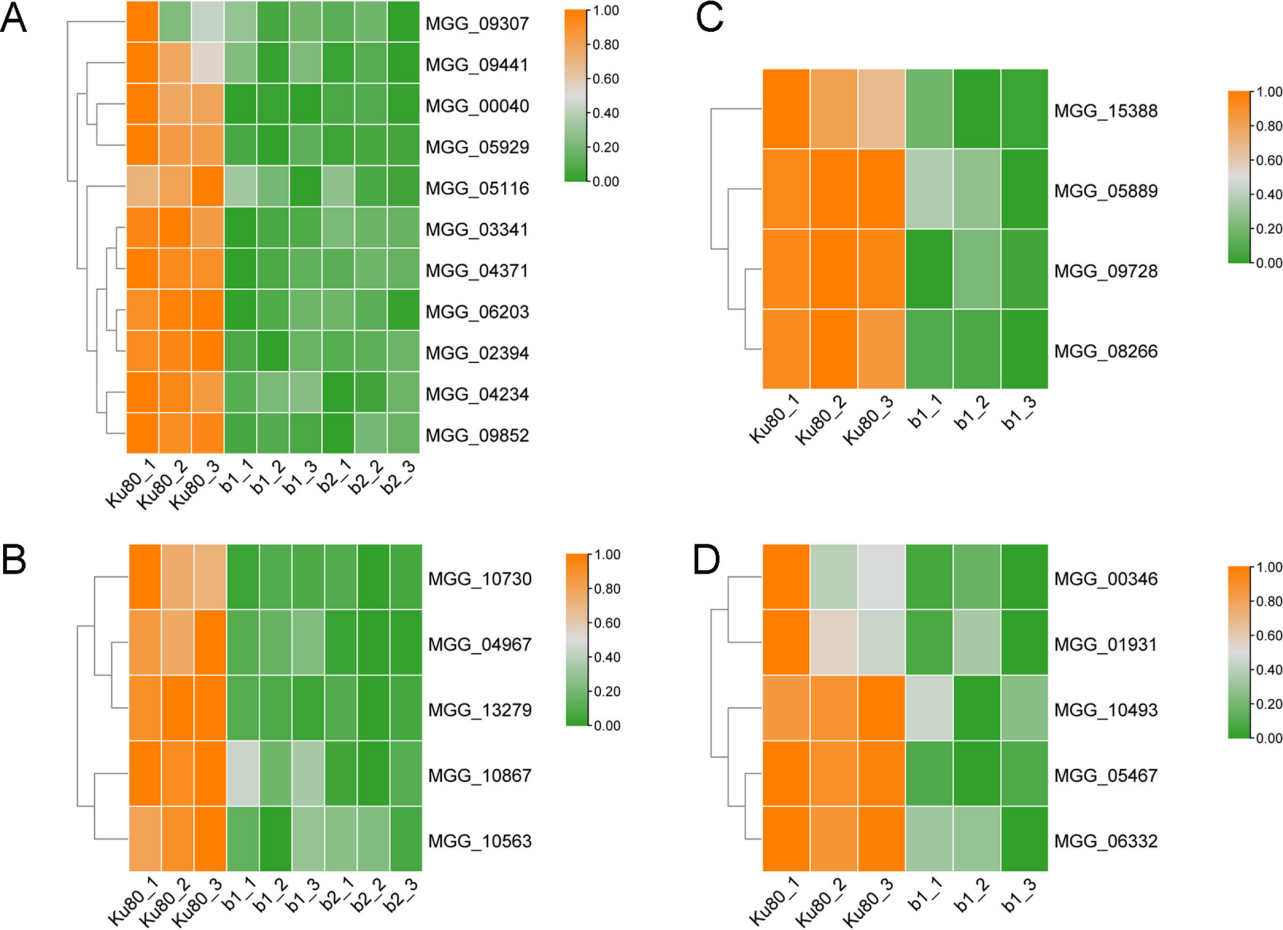
Expression of genes encoding transporters in the b1 and b2 mutants compared with background strain Ku80. (A) Expression of genes encoding carbohydrate transporters in both the b1 and b2 mutants compared with Ku80; (B) expression of genes encoding metal ion transporters in both the b1 and b2 mutants compared with Ku80; (C) expression of genes encoding lactose permeases and maltose permeases in the b1 mutant compared with Ku80; (D) expression of genes encoding metal ion transporters in the b1 mutant compared with Ku80. Genes showing similar patterns of expression are clustered.

We found that the expression of the genes encoding carbohydrate transporters was severely inhibited in the mutant. The expression of 11 genes was downregulated in both the b1 and b2 mutants. These genes were MGG_00040 and MGG_06203, encoding high-affinity glucose transporters; MGG_05929, MGG_04234, and MGG_09307, encoding hexose transporters; MGG_09852, encoding the sugar transporter STL1; MGG_03341, encoding plastidic glucose transporter 4; MGG_02394 and MGG_05116, encoding malic acid transporters; MGG_09441, encoding a tricarboxylate transporter; and MGG_04371, encoding a general α-glucoside permease (Fig. 8A and Table S7, sheets 2 and 4). In addition, b1 knockout affected the absorption and transport of lactose and maltose because of the downregulation of MGG_05889, MGG_09728, and MGG_15388, encoding lactose permeases, and MGG_08266, encoding the maltose permease MAL61 (Fig. 8C and Table S7, sheet 2). These results showed that the absence of the protein kinase MoCK2 decreased the ability of the mycelium to absorb and utilize sugar from the medium.

Besides free diffusion, the uptake of sugars also takes place by active transport. We also found that the expression of some ion transporter-encoding genes was downregulated in the mutants, such as MGG_05467, encoding sodium/nucleoside cotransporter 1; MGG_06332, encoding peroxisomal adenine nucleotide transporter 1; MGG_10867, MGG_04967, and MGG_10493, encoding zinc-regulated transporters; MGG_00346, encoding a phosphate transporter; and MGG_01931, encoding the siderophore iron transporter MirB (Fig. 8B and D and Table S7, sheets 2 and 4).

Compared with the downregulation of glucose transporters, the expression trend for genes encoding amino acid transporters in both mutants was not particularly obvious. Regulated amino acid transporters were found among the mutants’ up- and downregulated genes. MGG_08426, MGG_14203, and MGG_05833, encoding amino acid transporters, were downregulated in both mutants (Table S7, sheets 2 and 4). MGG_00289, encoding the amino acid permease Inda1, was downregulated. However, MGG_11327 and MGG_06036, which also encode amino acid permeases, were upregulated in the b1 mutant (Table S7, sheets 1 and 2). Similarly, the proline permease-encoding genes MGG_04216 and MGG_14937 were downregulated in the b1 mutant, but MGG_08548 was upregulated in the b2 mutant (Table S7). These results show that amino acid transporters had complex regulation in response to CK2 deletion.

### Genes known to directly interact with CKa are upregulated in the CKb mutants.

**FIG 9:**
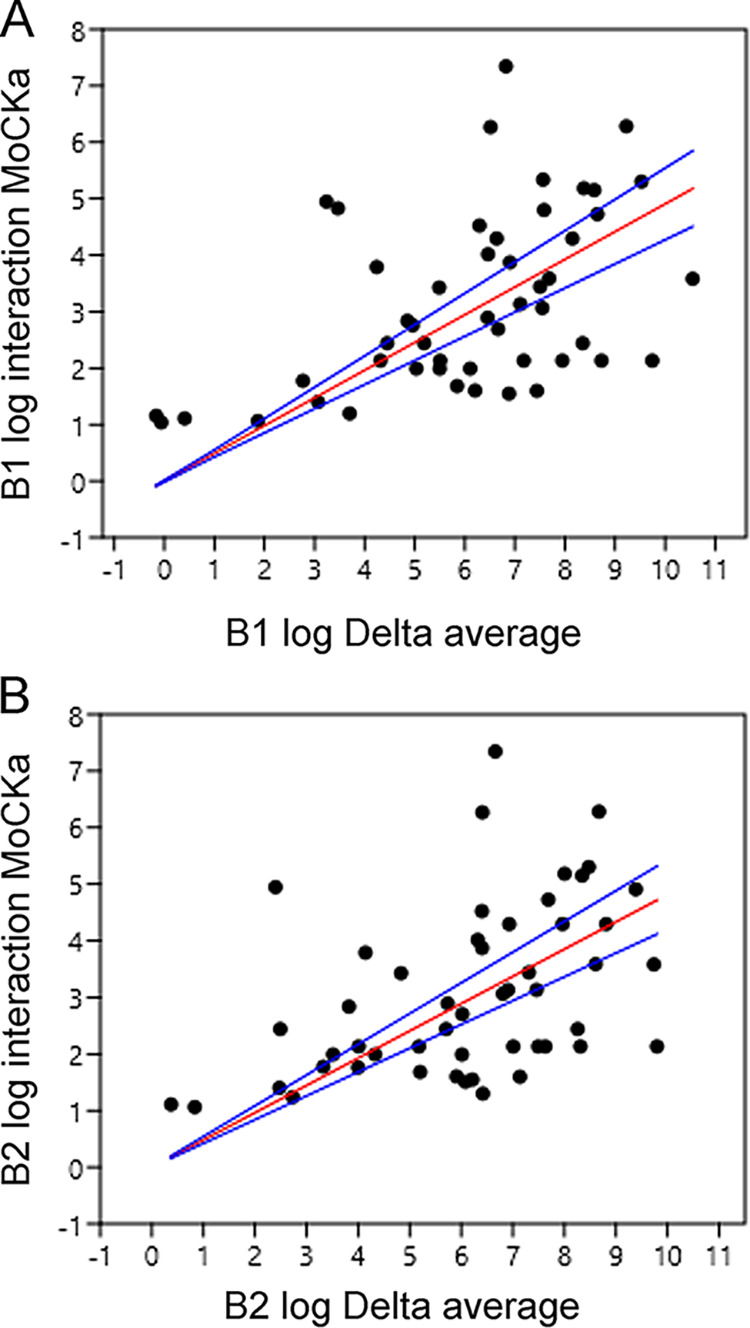
Upregulated genes also found in the CKa pulldown in a previous paper are correlated with the amounts of their encoded proteins found in the CKa pulldown. (A) Over a threshold, the upregulation of genes in the b1 mutant for proteins found in the CKa pulldown is generally correlated with the amounts of the respective proteins found in the pulldown (blue lines, ±95% confidence intervals for the red line [*P* = 0.00044 {not correlated}]). (B) Plot similar to the one in panel A but for the b2 mutant (blue lines, ±95% confidence intervals for the red line [*P* = 0.000849 {not correlated}]).

**FIG 10:**
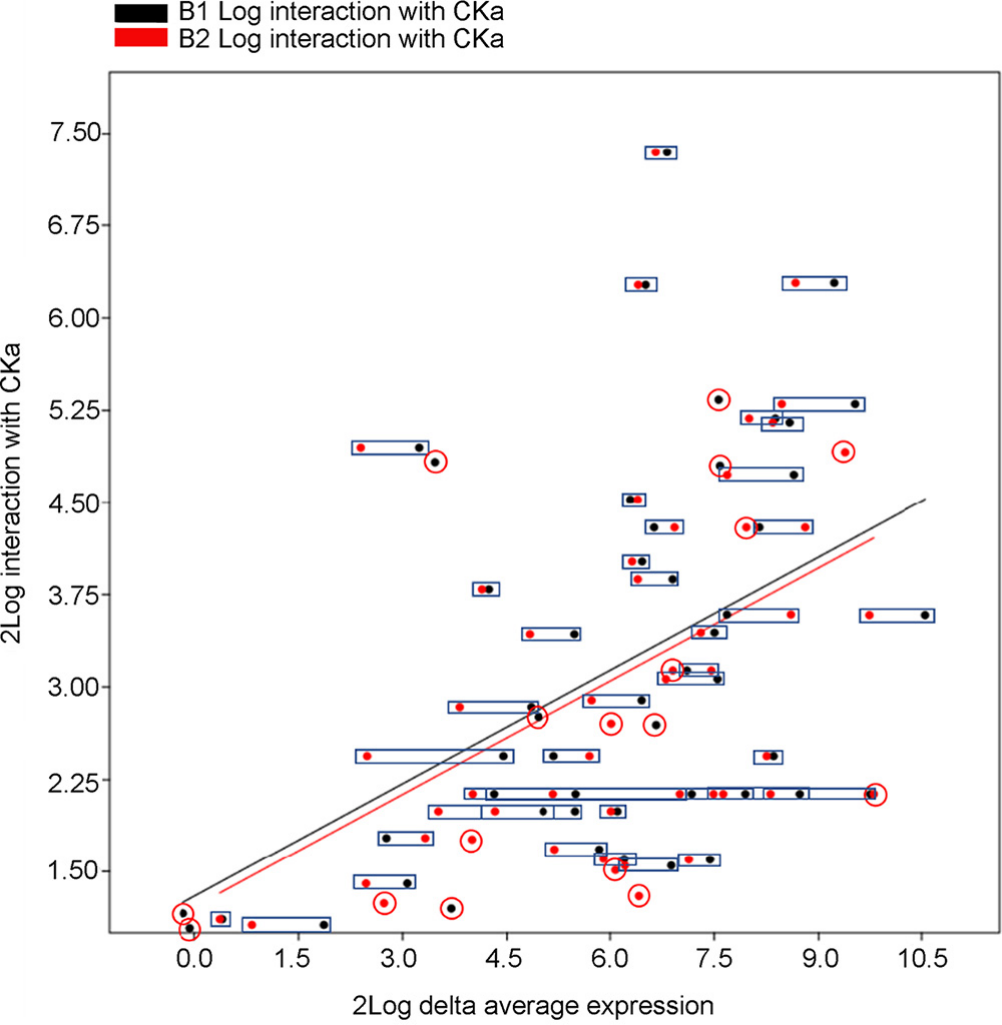
Combined plot of the data from Fig. 9A and B highlighting that the two relationships shown in Fig. 9 are very similar but with some genes being upregulated in only one mutant. Black dots, average regulation in the b1 mutant; red dots, average regulation in the b2 mutant. Black rectangles join regulations of the same gene in the b1 and b2 mutants. Red circles surround dots for genes significantly upregulated in only one of the mutants.

Of the significantly upregulated genes in the b1 and b2 mutants, 59 of the encoded proteins were found in the previous CKa pulldown (Tables S8 and S9). Since CKa is expected to phosphorylate these proteins and have a chaperone effect (16), a less efficient CK2 holoenzyme should chaperone these proteins, leading to the compensatory upregulation of their corresponding genes. Our results show that this is indeed the case (Fig. 9), where it is shown that when genes for proteins directly interreacting with CKa are upregulated more, the more they are found in the CKa pulldown. The relationship between these two measures is the same for the 60 significantly upregulated genes in the b1 and b2 mutants in that the two slopes for the fitted lines of the correlations are the same (Fig. 10). Interestingly, in addition, the expression of 8 genes is only up-regulated in the b1 mutant, and the expression of 9 genes is only up-regulated in the b2 mutant, indicating the differential involvement of CKb1 and CKb2 in regulating phosphorylation by CKa in the CK2 holoenzyme (Fig. 10 and Fig. S4).

**FIG 11:**
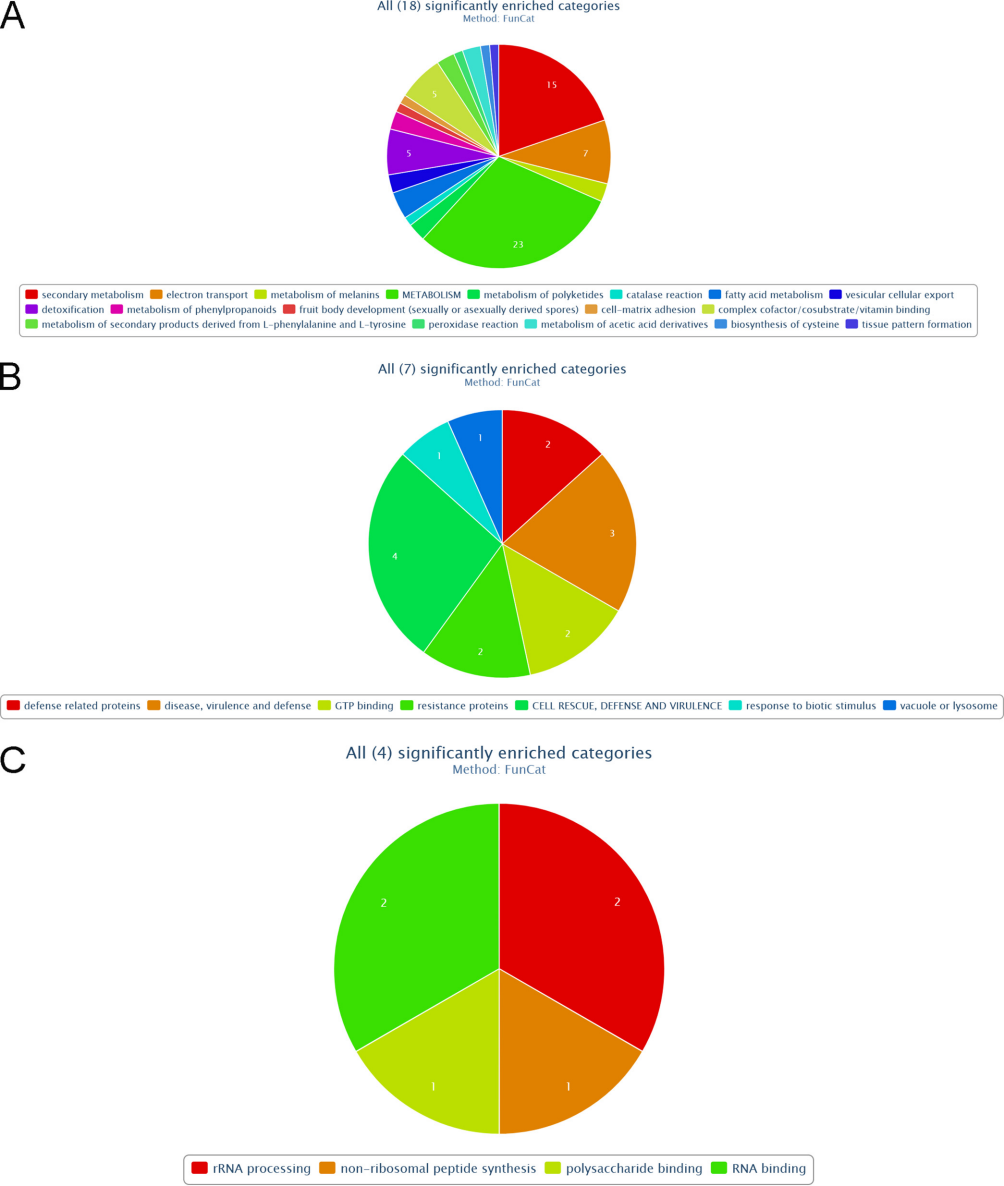
FunCat significantly enriched categories for significantly upregulated genes encoding proteins found in a CKa pulldown for both the b1 and b2 mutants (A), the b1 mutant only (B), and the b2 mutant only (C).

Genes for gene products interacting with CKa compensatorily upregulated in both b1 and b2 mutants were significantly enriched for genes involved in many categories important for plant infection and resistance to plant defenses (Fig. 11A and Fig. S5). The b1 mutant is enriched explicitly for gene categories of importance for plant pathogenesis (Fig. 11B and Fig. S6), and the b2 mutant is significantly enriched for genes necessary for translation (ribosome biogenesis) but also for making nonribosomal peptides like siderophores needed for iron uptake (Fig. 11C and Fig. S7).

## DISCUSSION

In previous research, we identified a vital protein kinase, MoCK2, that has a critical regulatory function for the growth, sporulation, and pathogenicity of M. oryzae (15). Moreover, MoCK2 pulldown experiments showed more than 1,300 hypothetical substrates (16). Therefore, we used RNA-seq technology to analyze the transcriptomes of background strain Ku80 and two mutants, b1 and b2, to analyze the MoCK2-regulating pathogenic mechanism in M. oryzae.

M. oryzae must absorb nutrients from the culture medium to maintain life activities. We collected the mycelia of the Ku80, b1, and b2 strains cultured in liquid complete medium (CM) for transcriptome sequencing. Like growth on solid medium, the growth rate of the b1 and b2 mutants in liquid culture was lower than that of Ku80. Therefore, we speculate that the deletion of MoCK2 may seriously inhibit the metabolism of M. oryzae.

The transcriptome analysis showed that an impaired CK2 holoenzyme caused significant expression changes in many genes in both mutants. KEGG and GO analyses showed that the genes with changed expression were enriched mainly in intracellular metabolisms such as carbohydrate metabolism, fatty acid metabolism, and amino acid metabolism. Further analysis showed that the deletion of MoCK2 seriously hindered the catabolism of substances in cells, directly reducing the concentration of acetyl-CoA in cells.

Acetyl-CoA is an important intermediate in various metabolisms. The impairment of MoCK2 appears to directly slow down pyruvate dehydrogenation and affects the conversion of pyruvate to acetyl-CoA. The catabolism of fatty acids in yeast and M. oryzae can be carried out in the peroxisomes, and acetyl-CoA is initially converted to acetyl-carnitine. Two carnitine acetyltransferases, Crat1 and Crat2, have been identified in M. oryzae, and the deletion of Crat1 especially has large effects on appressorium cell wall formation, the utilization of fats, and pathogenicity (27). Both Crat1 (MGG_01721) and Crat2 (MGG_06981) were significantly downregulated in the b1 and b2 mutants but around the −1-log_2_ fold change threshold level. For the b1 and b2 mutants, Crat1 was found to have log_2_ fold changes of −0.81 and −0.56, respectively (*P* = 3.33E−05 and 3.14E−03, respectively [no regulation]). For the b1 and b2 mutants, Crat2 was found to have log_2_ fold changes of −1.28 and −1.46, respectively (*P* = 1.92E−08 and 4.06E−11, respectively [no regulation]). This downregulation in the CKb mutants can partly explain the previously found phenotypic defects (15).

Thus, fatty acid β-oxidation and amino acid decomposition might be simultaneously reduced by the CK2 deletions, as the expression of related genes was significantly downregulated. The decreased expression of both the Crat1 and Crat2 genes encoding carnitine acetyltransferases and MGG_03418 encoding acyl-CoA dehydrogenase should affect β-oxidation and reduce the metabolism of acetyl-CoA. Amino acid degradation, especially serine degradation, is also an essential source of acetyl-CoA. Similarly, the expression of genes related to serine degradation in the mutants decreased significantly. In addition, the genes encoding a series of transporters involved in metabolism were downregulated, likely affecting the absorption and transport of sugar, pyruvic acid, and fatty acid, further slowing down the corresponding metabolic processes. These results fully demonstrate that MoCK2 participates in the metabolism of acetyl-CoA, as has also been shown for other organisms (28).

CKa interacts with CKb1 and CKb2 to form holoenzymes, and this interaction was confirmed in a pulldown experiment that also showed direct interactions with a large number of other proteins. We concluded in the previous study that CK2 phosphorylation with dephosphorylations likely contributed to the formation of protein-protein and protein-nucleic acid interactions through a chaperone-like mechanism dependent on CK2 phosphorylation (16). The lack of efficiency of the CK2 holoenzyme should consequently lead to a correlation between the absolute upregulation of the proteins and the protein abundance in the previous CKa pulldown, and this was indeed the case (Fig. 9 and 10). The up-regulated genes compensatory represented the proteins encoded by these genes should be lacking to be present in the correct shape and interaction. Interestingly, these genes were overrepresented for genes of importance for pathogenicity (Fig. 9; see also Fig. S5 to S7 in the supplemental material) and can further explain the b1 and b2 phenotypes found previously (15). Of these genes, many are predicted to encode mitochondrial proteins dependent on chaperone interactions to be imported into the mitochondria, and these were overrepresented among the CKa-interacting proteins (16). The destabilization of nonhydrophobic alpha helices that CK2 is capable of (16) should favor efficient mitochondrial protein import to the mitochondrion through the TOM and TIM complexes (29). In the absence of efficient import, these proteins would need to be compensatorily upregulated, which they are in both the b1 and b2 mutants (Fig. S8). These proteins were predicted to be mitochondrial using DeepMito (30). The predicted genes encoding proteins likely to be mitochondrial (Table S8) and present in the previous CKa pulldown are predicted to be located at the mitochondrial matrix (13), inner membrane (4), and outer membrane (3). There were more of these compensatorily regulated mitochondrial genes in the b1 mutant (19 of 20 total, with only 5 in b1) than in the b2 mutant (15 of 20 total, with only 1 in b2), further strengthening the results showing that the b1 mutant is more severely affected than the b2 mutant and in this case for genes encoding mitochondrion-located proteins.

Previous studies showed that the protein kinase MoCK2 accumulates at the mycelial septal pore, in the appressorium, and at the appressorium pore, possibly affecting cellular activities and material exchange between compartments. In addition, MoCK2 also accumulates in the nucleus, where CK2 can affect gene expression, transcription, the formation of ribosomes and other intrinsically disordered protein interactions (15). Such multiple functions were confirmed by the many changes in gene expression in both mutants. Our previous research detected many nuclear localization proteins interacting with MoCK2 through a pulldown experiment (16). Therefore, subsequent experiments will focus further on how MoCK2 affects the nucleus-localized proteins in response to physiological changes and during rice pathogenicity and on mitochondrial protein import into the mitochondria.

## MATERIALS AND METHODS

### Fungal strains and growth conditions.

All strains used in this study (background strain Ku80 and the b1 and b2 mutants) were stored on dry sterile filter paper, cultured in complete medium (CM) (0.6% casein hydrolysate, 0.6% yeast extract, 1% sucrose, 1.5% agar) at 25°C with shaking at 200 rpm, harvested by filtering through 3 layers of Miracloth (EMD Biosciences), washed, and frozen in liquid nitrogen.

### RNA extraction and RNA sequencing.

Total RNA was extracted from mycelia using the plant/fungal RNA purification kit from Sigma-Aldrich Trading Co. Ltd. (Shanghai, China). We used the Implen (Germany) P330 Ultra-Micro spectrophotometer to check the integrity and quantity of RNA. RNA with high integrity was used for library preparations. RNA was prepared from three biological replicates and used for independent library preparations.

High-throughput sequencing was performed on an Illumina HiSeq 2000 machine at Igenebook Biotechnology Co. Ltd. (Wuhan, China).

### Analysis of the quality of transcriptome data.

The genome of M. oryzae was downloaded from the Ensembl Fungi database (https://fungi.ensembl.org/). Clean reads were obtained from the raw reads with Trimmomatic, deleting low-quality sequences. The clean RNA-seq reads were aligned to the M. oryzae genome using hisat2 software (version 2.0.1-beta) (31). The alignment quality was calculated by the *Q*_20_, *Q*_30_, and GC content.

Nine sample hyphae for sequencing were collected from the background strain Ku80 and the b1 and b2 mutants. The transcriptomes of 9 samples were reconstructed by using Stringtie (version 2.0.4) and Gffcompare software (32, 33). The sequencing platform used was the Illumina HiSeq 2000 platform. The amount of data obtained by transcriptome sequencing of nine samples was between 7.1 Gb and 9.5 Gb. After filtering low-quality reads and adaptor sequences, ~6.9 Gb to 9.4 Gb of high-quality clean reads remained. The *Q*_20_ of clean reads ranged from 98.29% to 98.44%, and the *Q*_30_ ranged from 94.97% to 95.23%. Besides, the average GC content was about 56.53%, which was slightly higher than that of the reported genome of M. oryzae (see Table S1, sheet 1, in the supplemental material). The clean RNA-seq reads were aligned to the M. oryzae genome using hisat2 software (31). For the nine samples, the results showed that the lowest proportion of mapped reads was >83.27% (the highest was 93.64%) in clean reads. A total of 98% of the mapped reads were unique (Table S1, sheet 2).

We counted the effective reads compared to the genome according to the functional elements. We divided the genome into the coding DNA sequence (CDS), the 5′ untranslated region (UTR), the 3′ UTR, and intron and intergenic regions and counted the proportions of reads belonging to them. The results showed that more than 77% of the effective reads in 9 samples covered the CDS region, which met the requirements for data analysis (Fig. S1 and Table S1, sheet 3). The transcriptome data of 9 samples were analyzed by using Stringtie (32) and Gffcompare software (33) and compared with the reference genome data, and 3,023 new genes and 13,184 annotated genes were obtained (Table S1, sheets 4 and 5).

### Analysis of differences in gene expression among 9 samples.

After obtaining effective reads, we used featurecounts software (version 1.6.0) (34) to count the number of reads of the gene according to the annotation file for the M. oryzae genome. Because the amount of sequence for each sample is different, to horizontally compare the expression differences for the same gene among different samples, we needed to standardize the number of reads of the gene. The standardized method was the FPKM (fragments per kilobase of exon per million reads mapped) method (35). These results are shown in Table S1, sheet 6. Principal-component analysis (PCA) (36), which illustrates the intrinsic biological variation among samples, showed that samples from the same strains were grouped (Fig. S2A). The heat map, generated with the heatmap2 function in the gplots package, showed the similarity of gene expression patterns within the same strain, but there were significant differences between the background strain Ku80 and the two mutants (Fig. S2B).

In order to compare the differences in gene expression among different samples, we performed differential expression analysis using the R package edgeR (37). As a general rule, we considered genes with *P* values of <0.05 and absolute fold change values of >2 to be significantly differentially expressed genes (DEGs). These DEGs are shown in Table S1, sheets 7 to 12.

### Functional enrichment analysis.

In this study, we used TBtools to perform GO annotation, GO enrichment, and KEGG enrichment of DEGs between the b1 and b2 mutants and Ku80 (19, 20). In addition, we used FungiFun to perform FunCat and GO enrichment analyses of genes that were both upregulated and present in a previous CKa pulldown experiment since for that experiment, that Web tool had been used for the functional classification of the pulled-down proteins. The transcription abundance values for all genes were used to calculate the Euclidean distance between samples in the heat map analysis.

### Data availability.

The RNA-seq data described in this paper have been submitted to the Genome Sequence Archive at the National Genomics Data Center (NGDC) (https://ngdc.cncb.ac.cn/) under accession number CRA007756.
