# Supplementary material for: Transcriptome Analysis of Protein Kinase MoCK2, which Affects Acetyl-CoA Metabolism and Import of CK2-Interacting Mitochondrial Proteins into Mitochondria in the Rice Blast Fungus Magnaporthe oryzae
Source: Microbiol Spectr. 2022 Oct 18;10(6):e03042-22. doi: 10.1128/spectrum.03042-22 (PMC9769659; doi:10.1128/spectrum.03042-22)
Supplement: Supplemental file 1 — Fig. S1 to S8. Download spectrum.03042-22-s0001.pdf, PDF file, 0.7 MB [file spectrum.03042-22-s0001.pdf]

SUPPLEMENTAL MATERIALS

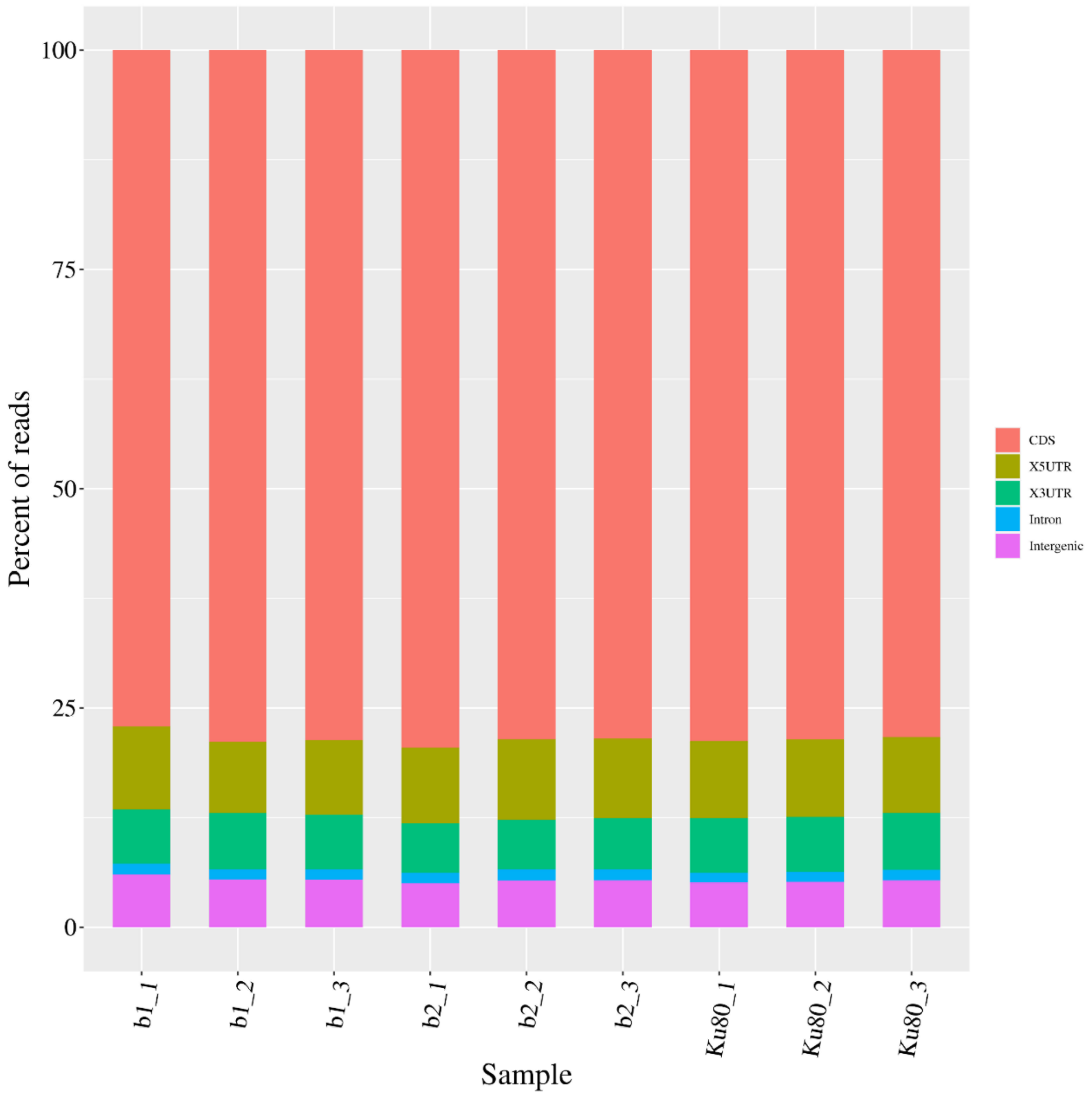

Fig. S1 Distribution table of reads on functional gene elements.

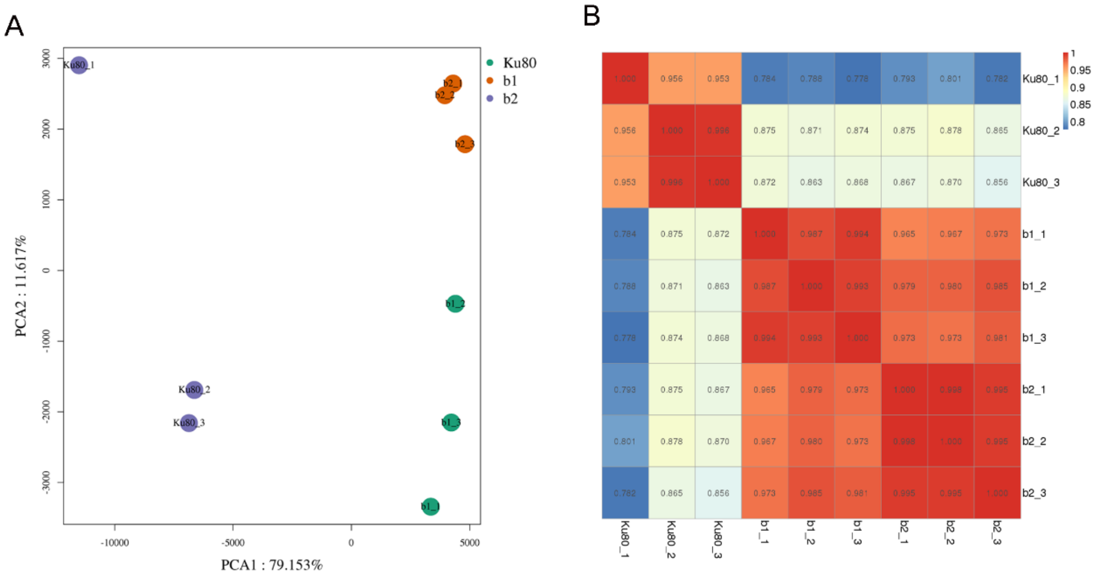

Fig. S2 Principal component analysis (PCA) (A)and heatmap (B)showed the gene expression in background strains Ku80, mutant b1 and b2

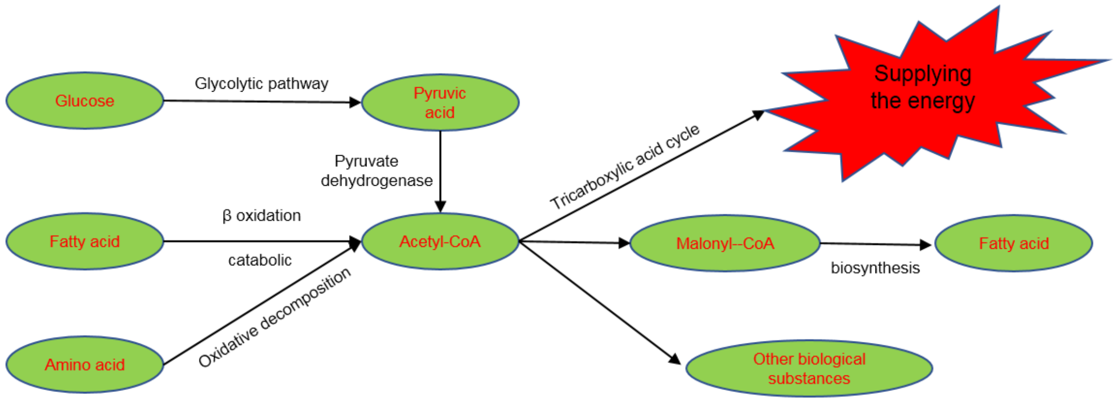

Fig. S3 The metabolic pathway of acetyl CoA production and utilization in cells

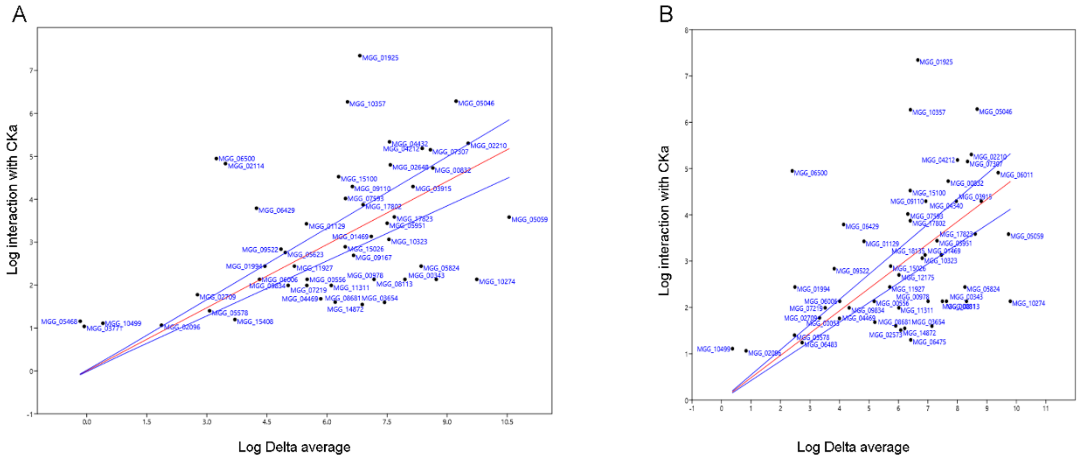

Fig. S4 A and B same figures as in Figure 9 but with MGG gene codes

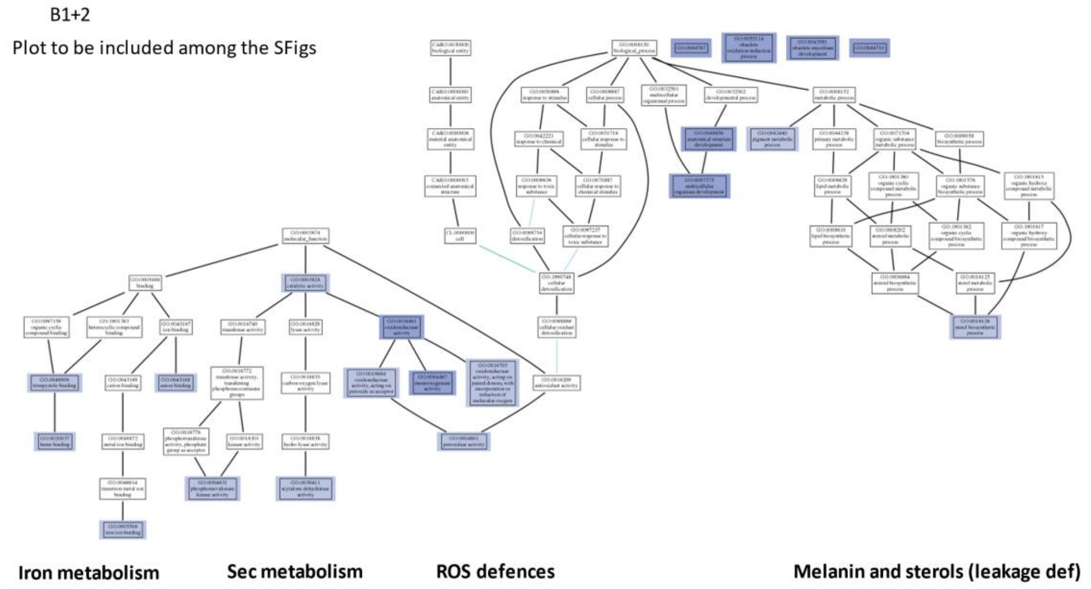

Fig. S5 GO categories significantly enriched among the genes upregulated in both b1 and b2 mutants that are also significantly present in the CKa pulldown from a previous study

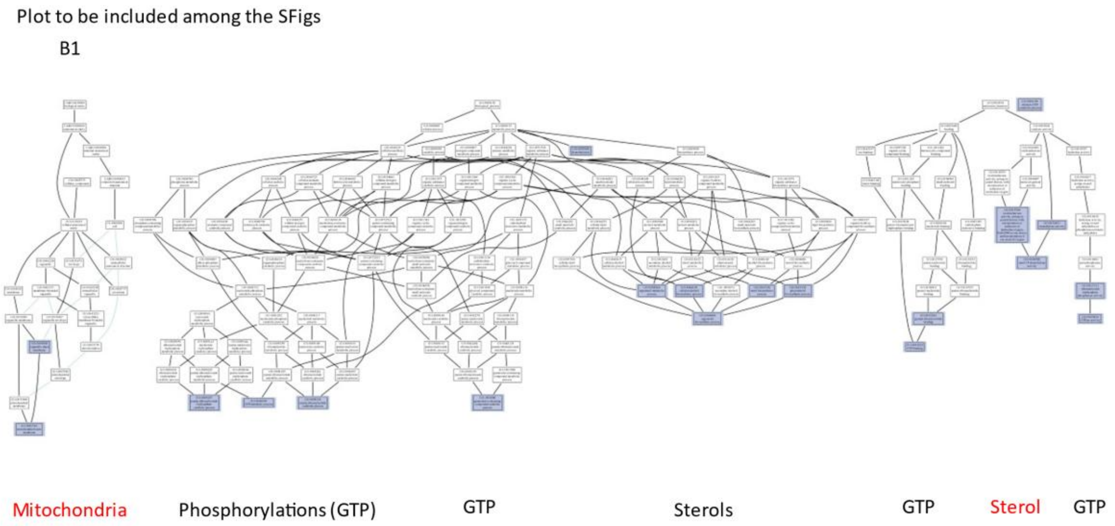

Fig. S6 GO categories significantly enriched among the genes upregulated in the b1 mutant that are also significantly present in the CKa pulldown from a previous study. Mitochondria and sterol metabolisms are specially marked since many mitochondrial proteins were found in the CKa pulldown, and CK2 destabilization of alpha helixes of mitochondrial proteins could be essential for the efficient transport of these proteins into mitochondria. Sterol metabolism is also affected, which indicates that the b1 mutant is more negatively affected by membrane integrity.

Plot to be included among the SFigs

B2

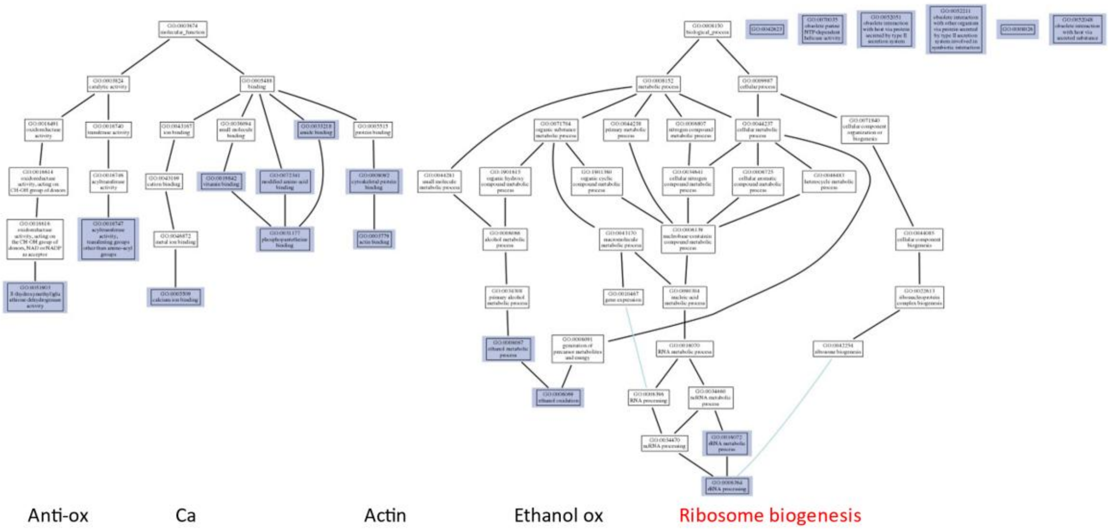

Fig. S7 GO categories significantly enriched among the genes upregulated in the b2 mutant that are also significantly present in the CKa pulldown from a previous study. Ribosome biogenesis is especially marked since the CKa pulldown was especially enriched for intrinsically disordered ribosomal proteins indicating that the b2 mutant might be more affected in protein synthesis rate than the b1 mutant. That should be less important for responses to plants but could be very detrimental for fast fungal immunity responses (38).

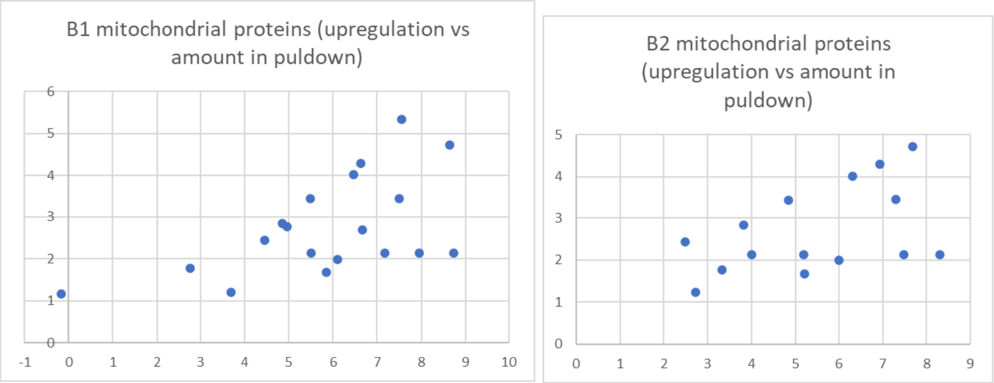

Fig. S8. Absolute upregulation of mitochondrial genes versus the amount of the corresponding gene product in a CKa pulldown point towards a compensatory upregulation of most of these genes
